# Natural Reproductive Management in Sarda Sheep: Use of Cryptorchids to Induce a Ram-Effect in Ewes Destined for Artificial Insemination

**DOI:** 10.3390/ani15233444

**Published:** 2025-11-28

**Authors:** Charbel Nassif, Laura Mara, Fabrizio Chessa, Marilia Gallus, Federico Melis, Ignazio Cossu, Antonello Ledda, Antonello Cannas, Maria Dattena

**Affiliations:** 1 Department of Agriculture, University of Sassari, 07100 Sassari, Italy; anledda@uniss.it (A.L.); cannas@uniss.it (A.C.); 2 Department of Animal Science, Agricultural Research Agency of Sardinia, 07100 Sassari, Italy; lmara@agrisricerca.it (L.M.); fchessa@agrisricerca.it (F.C.); mariliagallus@gmail.com (M.G.); federicomelis89@gmail.com (F.M.); icossu@agrisricerca.it (I.C.); mariadattena@gmail.com (M.D.)

**Keywords:** ovine, natural synchronization, teaser rams, artificial insemination, sustainable management

## Abstract

Cryptorchidism is a genital defect in which ram testicles fail to descend, causing azoospermia, while maintaining normal behavior towards females. We investigated whether cryptorchid rams can induce a ram-effect in ewes that would then be subjected to artificial insemination (AI). Therefore, ewes were isolated from any contact with rams for 6 weeks, then exposed to cryptorchid rams for 14 days. From day 15 to day 24, estrus was checked using a cryptorchid teaser four times daily (at 08:00, 12:00, 16:00, 20:00). Ewes detected in estrus were inseminated 24 h later. Experiment 1 included ewes (*n* = 31) all exposed to the cryptorchid ram-effect (CRE): 70.9% showed estrus, lambing rate after AI was 45.5%, and prolificacy was 1.40. Experiment 2 compared CRE (*n* = 80) with a control group with no prior exposure to males (*n* = 39). Estrus occurrence differed significantly (75.0% vs. 23.1%, respectively, *p* ≤ 0.001). Lambing rate from AI was 44.1% and prolificacy 1.27. These results show that cryptorchid rams effectively induce and synchronize estrus in Sarda ewes. AI fertility results on natural estrus following CRE yields outcomes comparable to those previously reported after hormonal synchronization for this breed.

## Simple Summary

Farmers relying on hormones to induce estrus in ewes for artificial insemination (AI) spend a lot of money, and this raises welfare and consumer concerns. This study explored a hormone-free, natural way to synchronize reproduction in Sarda sheep using cryptorchid rams. These are naturally sterile males, due to undescended testes, who still show normal behavior toward females. To prove their efficiency, ewes were first kept away from any contact with rams and then exposed to cryptorchids. The results showed that these males successfully induced and synchronized estrus in most ewes, with conception and lambing rates comparable to traditional hormone-based methods. This approach offers a safe, hormone-free, cost-effective, and sustainable alternative for managing sheep reproduction and artificial insemination naturally, supporting both genetic progress and welfare-friendly farming practices.

## 1. Introduction

Cryptorchidism is a genital defect in sheep, characterized by the partial or complete failure of testicular descent in affected rams [1,2,3,4]. Unilateral cryptorchids have only one retained testicle and are usually fertile. Bilateral cryptorchids have both testicles undescended and are sterile [3,4,5]. It is generally recommended not to use unilateral rams for reproduction, even if they are fertile, since it is an easily detectable and heritable trait [3,4,6]. Reports on the incidence of cryptorchidism vary between studies and breeds, fluctuating from 0.5 to 10.5% [5,7,8]. Since these animals are usually culled, their traits and possible uses have been scarcely studied. Some studies, after inducing cryptorchidism in adult rams reported a reduction in testicular size, degeneration of some structures, and a disruption of germ cell production. On the hormonal level, these animals maintained a normal testosterone concentration when compared to intact rams, while an increase in luteinizing hormone was noted [9,10]. This is consistent with the normal sexual behavior observed in cryptorchid rams towards females [11,12]. In fact, in a group of females, they have been shown to successfully identify and mount specifically those in estrus [13].

The Sarda sheep, one of Italy’s predominant breeds, has been part of a genetic selection program since the 1960s. The interest in this breed relies firstly on its quantitative and qualitative production (225 L in 180 milk days, with 6.0% fat and 5.3% protein [12]), and secondly on its semi-extensive rearing system, which is increasingly appealing to modern consumers. In addition, the Sarda breed displays distinct reproductive patterns that are relevant to flock reproductive management. This breed is mainly cyclic all year round with a brief anestrus season in late winter and early spring [14,15]. When rams are introduced to multiparous Sarda ewes at the end of the anestrus period towards May–June, they create a ram-effect synchronizing the ewes’ estrus [16,17].

The ram-effect is a well-documented technique that includes the sudden introduction of rams into a flock of ewes, after a period of separation, at the end of the anestrus period. The rams, through the combination of pheromones, visual and tactile cues, trigger the resumption of ovarian activity in ewes, as well as synchronizing estrus in the flock [18,19]. To perform artificial insemination (AI) instead of natural mating, the ram-effect has been performed using either intact rams with an apron or with the use of vasectomized rams [20,21,22,23]. While both techniques have proven efficient, they each present some difficulties. Using aprons on rams means heavily manipulating the males on a daily basis to attach the equipment and regularly removing it for cleaning. It might also cause lesions to the reproductive organs [24]. Vasectomizing rams can instead present animal welfare issues, besides the costs of the surgery and possible complications afterwards [25]. Therefore, using cryptorchids to induce a ram-effect on ewes that would then undergo an AI could present a practical, welfare-conscious alternative.

Genetic improvement relies heavily on selection as well as on the dissemination of genetics from highly valuable animals to achieve significant progress in a breed [26]. This is usually done by artificial insemination, a process in which selected ewes are typically synchronized by the exogenous use of hormones and then inseminated with diluted fresh semen from highly valued rams. This technique has proved efficient in genetic progress and selection schemes of breeds but has its own downsides [26]. The high costs of synchronization due to hormone prices and veterinary services present the first barrier to farmers [22,26]. AI in sheep results in low lambing rates; in the Sarda breed, these rates range from 30% to 60%, and prolificacy is between 120% and 130% [14,17,22,26]. Furthermore, there is a high animal welfare issue about the sourcing of hormones such as equine chorionic gonadotropin (eCG), which are produced from pregnant mares [27,28]. Lastly, modern consumers have a selective preference and support farmers and production systems that are hormone-free, to avoid any residues in food products [29,30]. All these reasons, in addition to the possibility of emerging new laws and regulations for the use of hormones in productive animals [22], indicate that we should search for alternative techniques to the use of hormones for estrus synchronization in ewes, thus allowing the farmers to perform AI and benefit from the gains it provides in genetic improvement, productivity, and animal health.

Given that bilateral cryptorchid rams are naturally sterile but maintain normal sexual behavior, we hypothesized that their introduction into ewe flocks would not pose a risk of impregnation while effectively inducing a ram-effect and synchronizing estrus in the flock for subsequent artificial insemination.

The aim of this study was to determine whether cryptorchid rams are an effective tool to induce a ram-effect and synchronize estrus in ewe flocks, and to evaluate the resulting fertility rates following artificial insemination.

## 2. Materials and Methods

To assess whether cryptorchid rams, aged between 1.5 and 6 years, born and raised on the Agris Sardegna experimental farm, could induce a cryptorchid ram-effect (CRE) in adult Sarda ewes (2 to 6 years of age), two experiments were performed: an initial preliminary trial and a second, larger-scale main trial conducted across two consecutive years as follows:

### 2.1. Experiment 1

To induce CRE, 4 unsheared cryptorchid adult rams were introduced to a flock of 31 ewes (ratio 1:8) in June at the experimental intensive farm of the University of Sassari at Ottava (Sassari, Sardinia, Italy; 40°46′30.89″ N, 8°29′14.57″ E).

**Figure 1:**
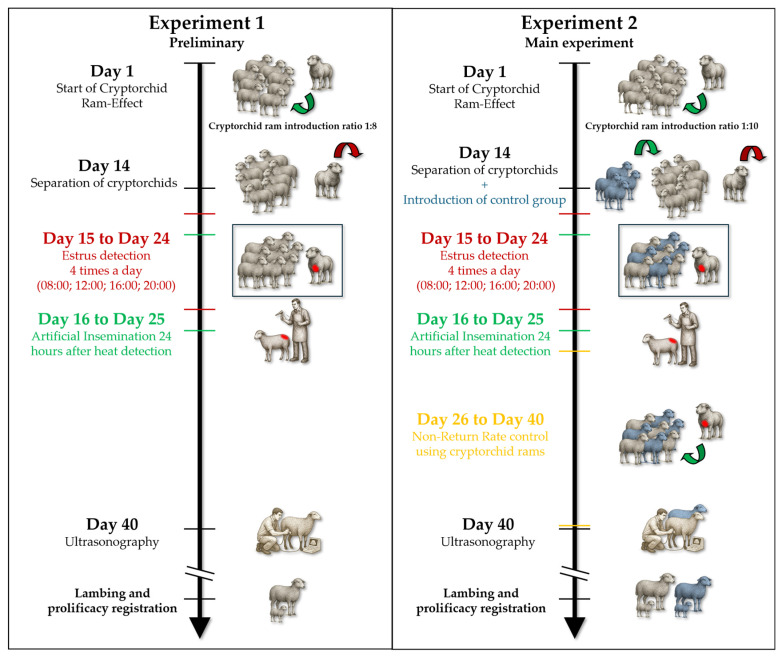
Flow chart of the experimental design for the preliminary (Experiment 1) and main (Experiment 2) trials using the cryptorchid ram-effect. Green arrows indicate the introduction of animals; red arrows indicate their removal. Blue animals represent the control group. The space between the two red lines indicates the estrus detection period; the space between the two green lines indicates the artificial insemination period; and the space between the two yellow lines indicates the non-return-rate monitoring period.

Cryptorchids were kept with the females for 14 days and then separated to better control estrus onset of ewes (Figure 1; A generative artificial intelligence tool (ChatGPT, OpenAI, San Francisco, CA, USA; model GPT-4/5.1) was used to generate illustrative images depicting the example sheep and human-animal handling positions used in the manuscript). Previous studies showed the ability of cryptorchid rams to detect females in estrus [13]. Thus, from day 15 to day 24, estrus detection was performed by introducing a group of 7 or 8 females inside a pen with one of the cryptorchid rams for 5 min. This procedure was repeated four times a day (at 08:00; 12:00; 16:00; 20:00) which allowed a precise determination of estrus onset during the day. The ram had a color marker on the chest. For each group, the behavior of both the ram and ewes was monitored by a technician. The females were considered in estrus when they showed typical behavior (ram-ewe seeking activity, fanning of the ewe’s tail, and ewe immobilization) and were mounted by the cryptorchid. The female in estrus was separated and then inseminated 24 h after estrus detection with cooled semen (4 °C). The animals were fed a total mixed ration. The body condition score (BCS) was recorded for all ewes and scored from 1–5 according to the method adapted by Molle et al. (2019) [31]. Ultrasonography was performed 40 days after AI to check the fertility rate.

### 2.2. Experiment 2

To induce the CRE, 8 unsheared cryptorchid adult rams were introduced to a flock of 80 ewes (ratio 1:10) in late May at the experimental semi-intensive farm of “Agris Sardegna” at Bonassai (Sassari, Sardinia, Italy; 40°40′22.74″ N, 8° 21′50.86″ E). Since in the preliminary study we noted that the cryptorchids were efficient, the ratio was reduced to 1:10. Cryptorchids were kept with the females for 14 days and then separated to better control estrus onset of ewes (Figure 1). Our objective was to make sure that the cryptorchids were responsible for estrus synchronization. Thus, on day 14, a control group of 39 ewes, which were not previously exposed to any contact with rams, was added to the group of 80 ewes, resulting in a total of 119 ewes.

From day 15 to day 24, estrus detection and insemination procedures were carried out as described in Experiment 1. After this period, females from the control group that did not exhibit signs of estrus (*n* = 28) were subsequently joined with fertile rams.

Ewes that underwent artificial insemination were monitored for the non-return rate (NRR). To assess this, two cryptorchid rams with chest color markers were placed with the females starting from the next day after insemination (Day 26). Marked ewes were checked and recorded twice daily (morning and afternoon).

All animals grazed for 4 h per day on natural pasture and received an additional 300 g of concentrate, along with hay provided ad libitum. Body condition score (BCS), on a scale from 1 to 5, was recorded for all ewes. Ultrasonography was performed 40 days after AI to assess fertility rate.

### 2.3. Semen Preparation

Semen was collected from rams with high genetic value, trained to ejaculate in an artificial vagina at the genetic center in Agris Sardegna–Bonassai. The quality parameters of all semen samples were immediately analyzed using a computer assisted sperm analysis (CASA) system (Ceros II, v1.13.7, Hamilton-Thorne, Beverly, MA, USA). Briefly, a 10 µL sample was diluted in 1 mL of saline solution (0.9% NaCl) and then evaluated under the microscope on a pre-warmed (38 °C) slide (Leja slides, 20 µm, IMV Technologies, L’Aigle, France). Five fields were selected and analyzed. Total motile sperm, progressive motility, and normal morphology were evaluated. The criteria to retain the semen and use it for AI were a minimum concentration of 3 × 10^9^ spermatozoa/mL, a total motility of at least 60%, progressive motility of at least 30% and no less than 70% of morphologically normal spermatozoa. When all the parameters were met, the semen sample was diluted to obtain 400 × 10^6^ spermatozoa/dose of 0.25 mL. The commercial medium used for dilution was OviXcell^®^ (IMV technologies, L’Aigle, France). After dilution, the semen sample was kept in the tube at 4 °C and used within 24 h of production.

### 2.4. Artificial Insemination

Twenty-four hours after estrus detection, the females were inseminated with the previously prepared cooled semen. The semen tube was gently mixed, and a 0.25 mL straw was filled. A standard cervical artificial insemination was then performed. In summary, the straw was loaded in the AI gun. The hind limbs of the female to be inseminated were elevated by an assistant, so that the animal was almost in a vertical position, allowing better visualization of the cervix. The inseminator inserted a speculum with a light source in the vagina, and once the cervix was in view, the AI gun was introduced until it reached the first cervical fold where the semen was released. It is noteworthy that, because of the convoluted anatomy of the Sarda sheep cervix, it was not possible to advance beyond the first cervical fold in any of the ewes. The female was gently lowered and then released.

### 2.5. Statistics

Statistical analysis of the results was performed using R software (version 4.5.1; R Core Team, Vienna, WIE, Austria) with RStudio (version 2025.9.0.387; Posit Software, Boston, MA, USA) [32]. Fisher’s exact test was used to analyze Estrus, NRR, Fertility, Lambing, and Prolificacy rates as well as for BCS, due to low animal numbers in some groups. Statistical difference was defined as *p* ≤ 0.05, results were expressed in terms of means ± standard error and percentages.

## 3. Results

### 3.1. Experiment 1

**Figure 2:**
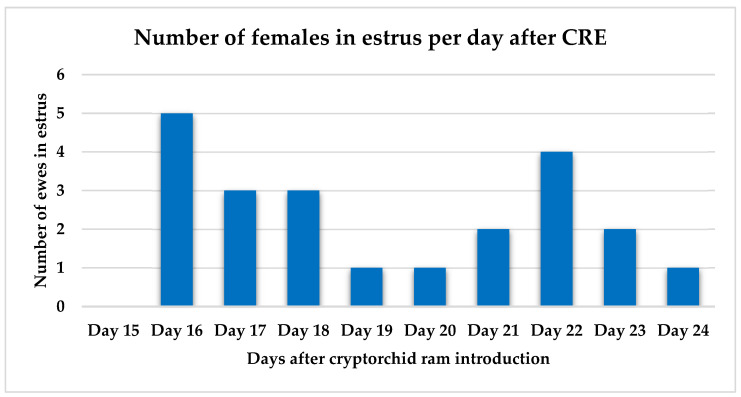
Distribution of ewes detected in estrus by cryptorchid rams from day 15 to day 24 after cryptorchid rams’ introduction.

**Figure 3:**
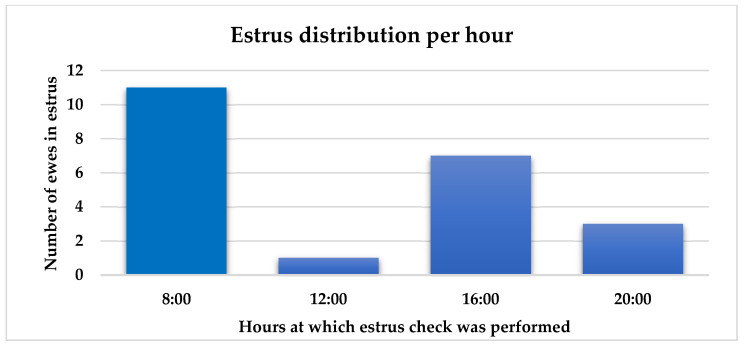
Number of ewes detected in estrus at four daily check intervals.

Out of 31 ewes that were exposed to the cryptorchid rams, 22 (70.9%) were detected in estrus between days 15 and 24 after ram introduction (Figure 2). Peak estrus occurred on days 16 and 22 after ram introduction. Eleven ewes were inseminated with cooled semen. Fertility and lambing rates were both 45.5% (5/11). Prolificacy rate was 1.4 lambs/ewe. Interestingly, most estrus events were detected in the morning at 08:00 (50.0%) followed by another peak in the afternoon at 16:00 (31.8%), while the lowest was at noon (Figure 3).

### 3.2. Experiment 2

**Figure 4:**
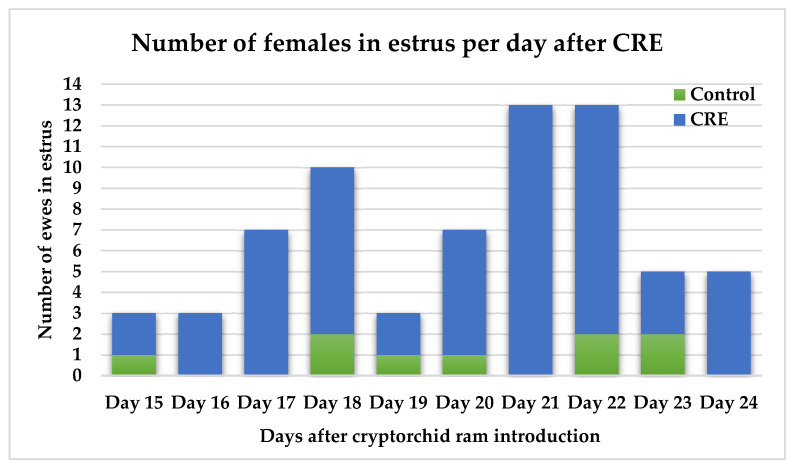
Distribution of ewes detected in estrus by cryptorchid rams from control and CRE groups from day 15 to day 24 after cryptorchid rams’ introduction.

**Figure 5:**
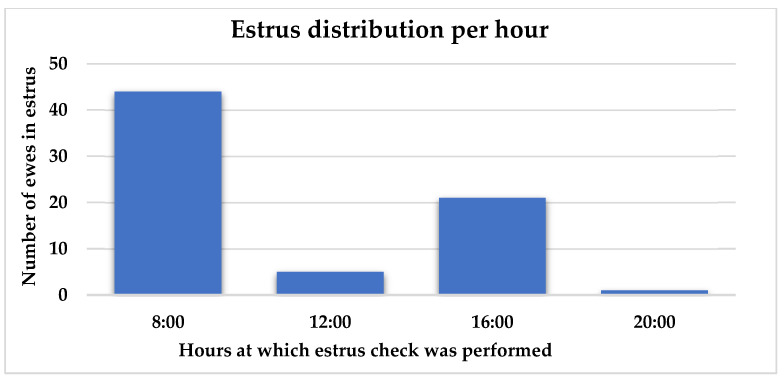
Number of ewes detected in estrus at four daily check intervals.

When comparing the CRE and control groups, 75.0% of the ewes exposed to cryptorchid rams (60/80) exhibited estrus signs between days 15 and 24, whereas only 23.1% of the control ewes (9/39) showed estrus signs during the same period. The results indicate a highly significant difference in the proportion of animals in estrus between the two groups (*p* ≤ 0.001). Peak estrus days after ram introduction were days 17–18 and 21–22 (Figure 4). Estrus distribution per hour of detection followed a similar pattern to Experiment 1, with most ewes (61.9%) having estrus signs at 08:00 followed by 29.6% at 16:00 (Figure 5).

The non-return rate for the control group was 66.7% and that of the CRE was 54.2%. Fertility results from ultrasonography of all inseminated ewes at 40 days was 50.0% (34/68) pregnancy rate (66.7% and 47.5% for control and CRE, respectively). Lambing rate was 44.1% for all the inseminated ewes (66.7% and 40.7% for control and CRE, respectively). Prolificacy was 1.27 lambs/ewe with 1 lamb per ewe for the control group and 1.33 lambs/ewe for the CRE. For the non-return rate, fertility, lambing and prolificacy, no significant difference was detected between both groups (Table 1).

We investigated the relationship of body condition score with fertility and lambing rates in individual animals. The animals in the experiment had a BCS range between 2 and 3.5. The animals with BCS 2 and 3.5 (2 and 3 ewes, respectively) were not included in the statistical analysis because of the low number of animals in these groups. Fisher’s exact test was performed for the statistical analysis to account for the low number of individuals in any group. No significant differences in fertility or lambing rates were seen across the BCS groups (*p* > 0.05).

It is noteworthy that the 28 females from the control group, which were not in contact with any rams prior to the experiment and were exposed to the cryptorchids for 10 days (estrus detection period), were then put with fertile rams. From these 28 ewes, 22 (78.6%) lambed after an average 166.8 (162–172) days from their first contact with cryptorchids, indicating that the cryptorchids also had a synchronizing effect on the estrus of this group of females.

## 4. Discussion

Naturally occurring cryptorchid rams have been rarely investigated in the scientific literature, and information on their general physiological characteristics remains limited. This limited interest may stem from their low practical value in commercial farming. Nevertheless, a few studies have reported that experimentally induced cryptorchidism does not markedly reduce testosterone concentrations compared to intact rams, and that the Leydig cells of cryptorchid males retain an enhanced steroidogenic capacity [9,10]. Because libido and courtship behaviors depend on adequate androgen signaling, and this signaling is not necessarily impaired in cryptorchid males, their sexual behavior may remain unaffected. Some husbandry practices induce cryptorchidism or create short-scrotum males to be used as sterile “teaser” rams. These same studies document that such males exhibit courtship and sexual behaviors comparable to those of intact rams [11,12].

The results of both Experiments 1 and 2 show a strong ability of cryptorchid rams to synchronize estrus in ewes, especially when compared with controls (*p* ≤ 0.001). Comparable results have been reported both for intact rams [23,33,34,35] and for vasectomized rams [22,36,37]. This is in accordance with these publications for the percentage of females that were synchronized (around 75%) and for the time required for them to exhibit signs of estrus (15–24 days after ram introduction). This suggests that although cryptorchids are infertile animals, they seem to have a sufficient pheromonal and hormonal production as well as a normal behavior toward females to stimulate cyclicity. The control group from Experiment 2 showed spontaneous estrus in some females amounting to 23.1%. This is a normal behavior of ewes that were not exposed to the ram-effect at the start of the breeding season. Reports on spontaneous estrus vary between 17% and 28% depending on the breed [38,39,40]. The combination of breed and seasonal period plays a major role in shaping the proportion of ewes in a flock that exhibit spontaneous ovulation at any given time [41]. One limitation of this study was the limited number of animals available in Experiment 1 and the management constraints which did not allow for a control group. The limitation in Experiment 2 was not including a separate group with intact rams to compare synchronization between the intact and cryptorchid groups.

The estrus occurrence trend showed two peaks, one at days 17–18 and the other at days 21–22, this pattern is commonly observed in ram-effect protocols. In detail, the sudden introduction of rams into the flock induces a pulsatile production of luteinizing hormone, which may lead to ovulation without estrus behavior; thus, estrus appears with the second ovulation 17–20 days later. The other peak is from ewes that experience a short 4–5 days luteal phase, ovulate without any estrus signs and then continue to have a normal cycle leading to late estrus behavior 21–24 days after ram introduction [18,19].

In the literature, the time of onset of estrus is rarely studied, and it probably differs by breed and geographical location. Similarly to our study, a report on Icelandic ewes indicates that estrus mainly occurs in the early hours of the day and in the afternoon, and less frequently at other times of the day [42]. On the other hand, in the Ile de France breed, the majority of estrus onset happened during the afternoon (17:00) [43]. This could be the result of high temperatures during the day, during which the animals tend to be less active.

As the ewes from Experiment 2 were monitored for another cycle (24 days) by placing two marked cryptorchid rams with the flock of inseminated ewes, we were able to monitor the non-return rate after insemination. Of all the inseminated ewes, 57.4% did not return to estrus. This dropped to a 50.0% pregnancy rate when ultrasonography was performed at 40 days post AI, and a lambing rate of 44.1%. It is usual to see a drop in the rates between NRR, pregnancy and lambing rate, due to an early embryonic loss common in AI programs involving Sarda sheep [20]. Possible reasons could be semen handling and quality, heat stress, or nutrition [44]. The lambing rates obtained from this natural method (45.5% and 44.1% from Experiments 1 and 2) are comparable to the results of AI (42%) when hormonal treatment is used in Sarda ewes [26]. Prolificacy also falls within averages as reported for the breed [14]. It is important to note that even if there is no statistical difference in prolificacy between both groups, the higher average of lambs per ewe from the CRE group might be due to the stimulation caused by the cryptorchids on the reproductive system, enhancing the follicular wave activity and thus having higher ovulation rates compared to the control group, which experienced spontaneous ovulation.

In our study, BCS groups did not significantly affect the results of either fertility or lambing rates, although higher percentages were seen for the BCS 2.75 group. It is still worth mentioning that most of the ewes (92.9%) were within the recommended BCS range (2.5–3.5) from previous research studies [45,46] and only a few were at the margins of the scale (2 ewes with a BCS of 2, and 3 ewes with a BCS of 3.5) which did not become pregnant and were excluded from the statistical analysis of BCS because of the low number of animals.

The ewes from the control group that were not inseminated were not kept for supervision after the 24th day from the start of the experiment. For managerial and practical reasons, this group was merged with a flock of ewes that were being mated by fertile rams. Interestingly, when collecting the lambing data, 78.0% of these ewes had lambed in a period of 162–172 days after their initial contact with cryptorchids. The range falls under the normal days from ram introduction to parturition reported in other studies [34,47]. This suggests that cryptorchid rams had indeed synchronized the cyclicity of these ewes even with a time-restricted contact (5–10 min of estrus check, 4 times a day for 10 days). This prospect could also suggest that stimulating females might necessitate a shorter period of exposure per day. Therefore, the same number of rams could stimulate a larger group of females if they are rotated among smaller subgroups while maintaining an adequate male-to-female ratio. Indeed, in goats it has been demonstrated that a buck ratio of 1:10 with a 4 h-per-day contact and a rotation into three groups of females efficiently stimulates estrus in does [48].

On the other hand, this technique, although it is easy, efficient, and cost-effective, might present some difficulties. The first is that it requires time and dedication from the farmer to monitor estrus onset within the flock. It also requires good communication with the semen production centers to be able to provide fresh semen when needed. Additionally, the insemination period would be spread over a few days instead of occurring at a fixed date and time as in hormonal treatment protocols. This issue, can be partly resolved by applying the ram-effect repeatedly over the years on the same farm [17]. Results over time confirm a shorter estrus period (4 days) and a higher percentage of animals showing estrus signs (96% after three consecutive years [20]). To simplify the CRE procedure, future studies could evaluate whether conducting estrus detection twice daily is as effective as the current four-times-per-day protocol. This adjustment may be applicable because most ewes exhibit estrus during the morning and late afternoon. Another practical alternative would be to inseminate only the ewes that are in the peak estrus concentration days (17–18 and 21–22 days after ram introduction), instead of inseminating the entire flock. This targeted approach could also be viable, as inseminating only part of the flock is generally sufficient for genetic improvement programs [49]. Under our conditions and based on our calculations using the 1:10 ratio, synchronizing 10 ewes using hormones for artificial insemination (including the cost of hormones, veterinary services, and discarded milk during the synchronization period) was 1.5-fold more expensive than maintaining one cryptorchid ram on the farm for an entire year, including feed and management costs.

This technique could be considered a viable solution for farmers, especially for organic farmers, wanting to apply a reproductive program, whether it is AI or ‘controlled natural mating’ (the act of mating a single desired ram with more females, enabling registration of offspring paternity [49]) to enhance the genetics of a flock. Another advantage is the ability of farmers to perform the whole process on their own if they are trained properly, since there is no need to buy any pharmaceutical products.

## 5. Conclusions

This study demonstrates that the introduction of cryptorchid rams is a safe and natural tool to significantly synchronize estrus in a flock of ewes at the end of the anestrus period. This low-cost technique can be used to group estrus onset without the need to administer any hormonal treatment. This synchronization of estrus can help farmers control the reproduction of the ewes either by performing artificial insemination with ram semen of proven high genetic value or by using it for ‘controlled natural mating’. This method has some disadvantages, such as being laborious and time-consuming, especially when the animals are monitored in small groups and four times per day. Additionally, farmers should plan ahead by keeping and raising cryptorchids until they reach adulthood before their use for the ram-effect. Future studies should investigate ways to simplify the method, maintain high estrus-synchronization efficiency, and to extend semen shelf life, thus reducing the current limitations of this technique and making it more field-appropriate. This would give additional incentives to farmers to apply this technique on their farms.

## Figures and Tables

**Table 1:** Results of the CRE vs. control group ± SE showing estrus as well as non-return rate, fertility, lambing and prolificacy rates after insemination.

Group	Ewes in Estrus (%)	NRR (%)	Fertility Rate (%)	Lambing Rate (%)	Prolificacy Rate (%)
Control (*n* = 39)	23.1 ± 0.07	66.7 ± 0.08	66.7 ± 0.08	66.7 ± 0.08	100 ± 0.00
Cryptorchid Ram-Effect (*n* = 80)	75 ± 0.05 *	54.2 ± 0.06	47.5 ± 0.06	40.7 ± 0.06	133.3 ± 0.05

* Indicates a *p*-value ≤ 0.001.

## Data Availability

The data presented in this study are available on request from the corresponding author.

## Abbreviations

The following abbreviations are used in this manuscript:

AI	Artificial Insemination
CRE	Cryptorchid Ram-Effect
eCG	Equine Chorionic Gonadotropin
NRR	Non-Return Rate
